# Factors Affecting Spatial Variation of Annual Apparent Q_10_ of Soil Respiration in Two Warm Temperate Forests

**DOI:** 10.1371/journal.pone.0064167

**Published:** 2013-05-22

**Authors:** Junwei Luan, Shirong Liu, Jingxin Wang, Xueling Zhu

**Affiliations:** 1 The Research Institute of Forest Ecology, Environment and Protection, Chinese Academy of Forestry, Key Laboratory of Forest Ecology and Environment, China's State Forestry Administration, Beijing, PR China; 2 West Virginia University, Division of Forestry and Natural Resources, Morgantown, West Virginia, United States of America; 3 Baotianman Natural Reserve Administration, Tuandong, Chengguan Town, Neixiang County, Henan Province, PR China; DOE Pacific Northwest National Laboratory, United States of America

## Abstract

A range of factors has been identified that affect the temperature sensitivity (Q_10_ values) of the soil-to-atmosphere CO_2_ flux. However, the factors influencing the spatial distribution of Q_10_ values within warm temperate forests are poorly understood. In this study, we examined the spatial variation of Q_10_ values and its controlling factors in both a naturally regenerated oak forest (OF) and a pine plantation (PP). Q_10_ values were determined based on monthly soil respiration (R_S_) measurements at 35 subplots for each stand from Oct. 2008 to Oct. 2009. Large spatial variation of Q_10_ values was found in both OF and PP, with their respective ranges from 1.7 to 5.12 and from 2.3 to 6.21. In PP, fine root biomass (FR) (R = 0.50, *P = *0.002), non-capillary porosity (NCP) (R = 0.37, *P = *0.03), and the coefficients of variation of soil temperature at 5 cm depth (CV of T_5_) (R = −0.43, *P = *0.01) well explained the spatial variance of Q_10_. In OF, carbon pool lability reflected by light fractionation method (*L_LFOC_*) well explained the spatial variance of Q_10_ (R = −0.35, *P = *0.04). Regardless of forest type, *L_LFOC_* and FR correlation with the Q_10_ values were significant and marginally significant, respectively; suggesting a positive relationship between substrate availability and apparent Q_10_ values. Parameters related to gas diffusion, such as average soil water content (SWC) and NCP, negatively or positively explained the spatial variance of Q_10_ values. Additionally, we observed significantly higher apparent Q_10_ values in PP compared to OF, which might be partly attributed to the difference in soil moisture condition and diffusion ability, rather than different substrate availabilities between forests. Our results suggested that both soil chemical and physical characters contributed to the observed large Q_10_ value variation.

## Introduction

Soils are the largest carbon pool in the terrestrial ecosystem, estimated to contain almost three times as much carbon as the atmosphere between the depths of 0–300 cm of soil [1], [2]. This value is much higher if northern permafrost regions are also considered [3]. Annual CO_2_ efflux from soil respiration (R_S_), the second largest terrestrial carbon flux, is ten times higher than CO_2_ efflux from fossil burning [4], [5]. R_S_ is also probably the least well constrained component of the terrestrial carbon cycle [6]. Thus, the response of R_S_ to climate change, which usually is called apparent temperature sensitivity of R_S_ (Q_10_ value) and estimated based on empirical functions, is of importance in predicting possible feedbacks between the global carbon cycle and the climate system [7]. Recently, the efficiency and accuracy of R_S_ estimation based on apparent Q_10_ values and the method used to estimate Q_10_ values [7], [8], has been widely debated [9]. Nevertheless, empirical response functions are still a valid method to derive annual estimates of R_S_ based on specific field measurements (e.g. Savage et al. [10]), particularly when it is not limited by water content and the simulation is made through interpolation rather than extrapolation [11].

The Q_10_ of R_S_ has been a focus of R_S_ research and is widely reported in the literature. Soil moisture condition has been suggested to be a factor that affects Q_10_ [12]–[14]. However, a positive [14] or a topographic position dependent [13] relationship between soil moisture and Q_10_ has been reported. Davidson and Janssens [15] pointed out that soil moisture could exert a secondary effect on apparent Q_10_ due to its interaction with substrate availability [16]. The seasonal change in autotrophic respiration, which is driven by the strong seasonality in tree below ground C allocation, could also influence the variability in apparent Q_10_ values [17], [18]. Thus annual and seasonal variations of Q_10_ values have been widely reported [14], [19]. Furthermore, the relationship between soil organic matter (SOM) quality and temperature sensitivity of organic matter decomposition has been extensively studied recently [7], [8]. Whether SOM of different quality has similar [20]–[22] or different temperature sensitivities has also been debated [23]–[25].

The variability of temperature sensitivity among ecosystems has been reported, accounting for substrate quality [23], climate factors [26], or different range of temperature used to estimate Q_10_ values [27]. Mahecha et al. [28] found a global convergence in the temperature sensitivity of respiration at the ecosystem level, but high spatial variation of temperature sensitivity exists within plots [14], [29]. Spatial variation of R_S_ has been discussed, e.g., in boreal forest [30]; tropical rainforest [31]; as well as savanna ecosystem [32]. However, direct field evidence of factors affecting the spatial variation of apparent Q_10_ values within plots has not been fully investigated, and it is still ambiguous whether variation is attributed to the spatial distribution of SOM quality or soil microclimate.

In this study, both a natural regenerated oak forest (OF) and a nearby artificially regenerated pine plantation (PP) were chosen in warm temperate China, to determine characteristics of spatial variability of apparent Q_10_ values within plot at locations in a 10 m×10 m grid based on R_S_ field measurements. Our specific objectives were to 1) identify the spatial variation of Q_10_ values in both OF and PP; and 2) determine factors correlated with spatial variability of Q_10_ values within each plot.

## Materials and Methods

### Study Sites and Experimental Design

The study sites were located at the Forest Ecological Research Station in the Baotianman Natural Reserve (111°47′–112°04′E, 33°20′–33°36′N), Henan Province, PR. China. Baotianman Natural Reserve Administration (Neixiang County, Henan Province) issued the permission for our experimental sites. The average elevation is 1400 m, with an annual mean precipitation and air temperature of 900 mm and 15.1°C, respectively. Precipitation occurs mainly in summer, accounting for 55–62% of the annual total [33]. Upland soils are dominated by mountain yellow brown soils (Chinese classification). The OF stand was dominated by *Quercus aliena* var. *acuteserrata*, while the nearby PP stand was dominated by *Pinus armandii* Franch (for detailed information of these two stands see Luan et al. [34]). No intensive management was conducted in the PP since its establishment. One 40 m×60 m study plot was delineated in each stand with an average slope of <8°. Within each plot, a 10 m×10 m square grid was then placed and 35 subplots (1 m×1 m) were positioned at each intersection of the grid. PVC collars (19.6 cm inside diameter) were installed at each subplot in September 2008 and were kept on the site throughout the study period.

### Soil Respiration, Microclimate Measurements, and Q_10_ Calculation

Soil respiration measurements were conducted for a total of 12 (OF, measurement on 19 May, 2009 was canceled due to rain event) and 13 (PP) measurement campaigns using a Li-8100 soil CO_2_ flux system (LI-COR Inc., Lincoln, NE, USA), from October 2008 to October 2009 avoiding snow cover period (9 and 17 Oct., 1 and 11 Nov. of 2008; 19 Mar., 7 and 17 Apr., 19 May., 2 and 23 Jun., 2 Aug., 19 Sept., and 19 Oct. of 2009). Sampling was performed between 9∶00 and 15∶00 (GMT +8∶00). Soil temperature at 5 cm (T_5_) was measured adjacent to each respiration collar with a portable temperature probe provided with the Li-8100. Soil volumetric water content (SWC) at 0–5 cm was measured with a portable time domain reflectometer MPKit-B soil moisture gauge (NTZT Inc., Nantong, China) at three points close to each chamber. We avoided early morning and post-rain measurements to reduce the possible effect of rapid transition on the soil respiration rate during the observations.

An exponential equation (Eqn (1)) was used to describe the temporal relationship between R_S_ and T_5_ for each subplot (n = 12 for OF; or 13 for PP):
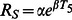
(1)where *R_S_* is soil respiration; *T_5_* is the soil temperature at 5 cm depth; and *α* and *β* are fitted parameters. The temperature sensitivity parameter, Q_10_ of each subplot was calculated as:



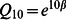
(2)Our analysis showed that one measurement fewer for OF compared to PP do not have significant impact on Q_10_ estimation (data were not shown).

The number of samples required to estimate the Q_10_ of R_S_ of each stand at the 10% or 20% of its actual value at the 95% probability level was obtained using Eq. 3 described by Hammond and McCullagh [35]:
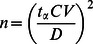
where *t_α_* is Student’s t with degrees of freedom (α = 0.05), *CV* is the sample coefficient of variation derived from data obtained for this study, and *D* is allowable error of field sampling process.

### Soil Properties, Root Biomass, and Carbon Pool Lability

Five soil samples were collected from the top 5 cm depth of the mineral soil next to each chamber using 100 ml (50.46 mm diameter, 50 mm height) sampling cylinders in August, 2009. Three soil samples were combined and used for mass-based measurements of soil organic carbon (SOC), total nitrogen (TN), and light fraction organic carbon (LFOC). The remaining two cylinder samples were used for analyses of bulk density (BD), total soil porosity (TP), capillary porosity and non-capillary porosity (NCP) on the basis of soil water-retention capacity [36]. Light fraction soil organic matter at a depth of 0–10 cm was obtained by the density fractionation method proposed by Six et al. [37], but with a modification using CaCl_2_ solution (density of 1.5 g ml^−1^; Garten et al. [38]). Bulk-soil and light-fraction organic carbon contents were determined by the wet oxidation method with 133 mM K_2_Cr_2_O_7_ at 170–180°C [39]. In August 2009, roots were extracted from 0–30 cm fresh soil samples by two cores (10 cm diameter) located close to the collars. The samples were washed; coarse (>5 mm), medium (2–5 mm), and fine (<2 mm) roots were manually separated and then their dry biomass (70°C, 24 hours) was measured. We found that stand structure parameters (total basal area, maximum DBH for trees within 4 m (radius) of the measurement points) well explained the spatial distribution of fine root biomass [34], which indicated that the spatial pattern of fine root biomass is comparably stable, because stand structure is relatively stable for an ecosystem in a given time. The leaf area index (LAI) was measured above each subplot using hemispherical photographs with WinSCANOPY (Regent Instruments Inc., Quebec, Canada) in August 2009.

The term ‘lability’ of SOC was defined as the ratio of the oxidized to non-oxidized SOC [40]. We applied this definition to the density fractionation method, and calculated subplot carbon pool lability (*L_LFOC_*) as described by Luan et al. [41]:
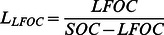
(3)


LFOC is the light fraction organic carbon and SOC is the soil organic carbon.

### Statistical Analysis

Descriptive statistics (mean, range, standard deviation (SD) and coefficient of variation (CV)) were used to show the characteristics of the spatial variability of R_S_, Q_10_, and soil parameters. Variogram computations were also performed to determine the strength and scale of the spatial variability of Q_10_ and soil parameters. The spatial variability was quantified by the semivariance (*γ* (h)). The semivariance of any parameter z is computed as:
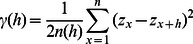
(4)where *n* (*h*) is the number of lag pairs at distance intervals of *h* and *z_x_* and *z_x+h_* are the values of the variable z at *x* and *x+h*, respectively. Plotting *γ*(h) against h gives the semivariogram, which will exhibit either purely random behavior or systematic behavior described by a theoretical model (linear, spherical, gaussian or power law distribution). The nugget, sill, range and structural variance (Q) parameters were obtained from the model with the best fit to the semivariance data. Geostatistical analyses were performed with GS+ (Geostatistics for the Environmental Sciences, v.5.1.1, Gamma Design Software, Plainwell, MI).

**Figure 1:**
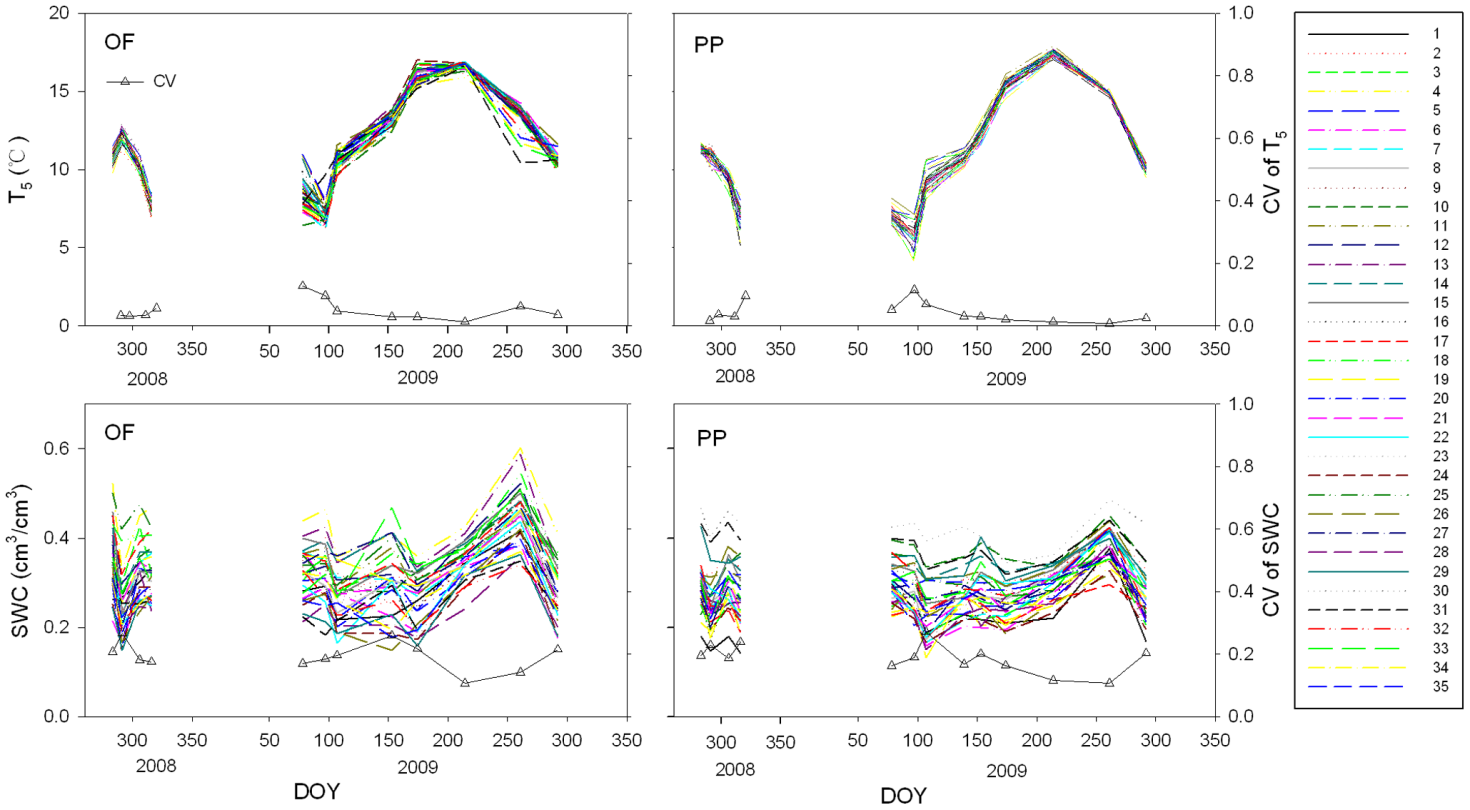
Semivariograms of L_LFOC_ (a, f), FR (b, g), SWC (c, h), NCP (d, i), and Q_10_ (e, j) in 10-m grid squares of OF (left panel) and PP (right panel), respectively. Model for SWC are exponential models. The SWC were averaged over the 12 (OF) or 13 (PP) measurement campaigns.

Pearson correlations were performed to assess factors (soil moisture, seasonal CV of T_5_ and SWC, LFOC, *L_LFOC_*, FR, NCP) controlling spatial variation of Q_10_ values among subplots for each forest (n = 35) or pooled data of two forests (n = 70). Geostatistical analyses showed that Q_10_ values and soil parameters were spatially independent (Fig. 1). This allowed us to treat our measurement locations as independent samples for inferential statistics. Therefore, general linear models (GLM) were employed to examine the effect of forest type on Q_10_ values, where *L_LFOC_*, FR, SWC (averaged over 12 or 13 measurement campaigns), and NCP were included in the model as co-variables, respectively. Statistical analyses were conducted using SPSS version 13.0 (SPSS Inc., Chicago, USA).

## Results

### Microclimate and Soil Parameters Variance within Plots

**Figure 2:**
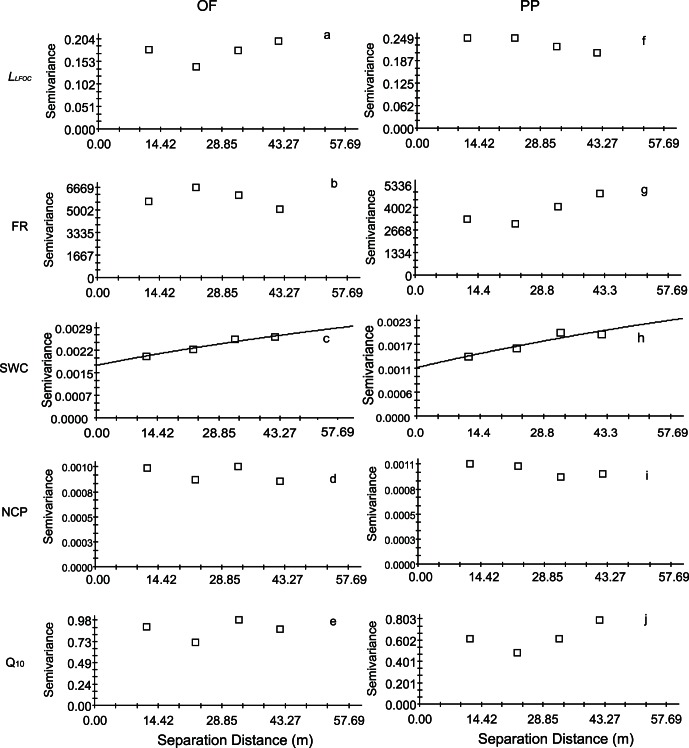
Seasonal pattern of T_5_ (up panel) and SWC (lower panel) for OF (left panel) and PP (right panel) for each subplot, as well as the seasonal pattern of the CV (up triangle) of T_5_ and SWC among subplots.

All the subplots experienced similar seasonal fluctuations of T_5_ and SWC (Fig. 2). High spatial variation of SWC was found in all measurement campaigns (Fig. 2), with the CV of SWC ranging from 10.7% to 27.2% for PP and from 10.7% to 26% for OF (Fig. 2). Soil carbon and nitrogen contents at 5 cm depths, the C/N ratio, soil bulk density, light fraction organic carbon, fine root biomass and soil carbon pool lability (*L_LFOC_*) for the OF and PP showed high spatial variation in the stand (Table 1). The semivariograms of *L_LFOC_*, FR, and NCP showed no change in semivariance with distance, indicating that they had no spatial autocorrelation in this scale (Fig. 1 a, b, d, f, g, i). Although averaged SWC had moderate spatial dependency, the ranges and sills observed were not precisely determined because the ranges were larger than the effective range of 43.27 m, which is equal to 60% of the maximum lag in the 10-m grids (Fig. 1 c, h).

**Table 1:** Statistical analysis of soil parameters, fine root biomass, soil respiration rate, Q_10_ values, and carbon pool lability (*L*_LFOC_) for the oak forest and pine plantation.^a^

Parameters	Oak forest				Pine plantation			
	mean	S.D.	Range	CV	mean	S.D.	Range	CV
R_S_ (µmolm^−2^s^−1^)	2.12	0.58	1.16–4.17	0.27	2.01	0.44	1.07–3.16	0.22
Q_10_	3.80	0.95	1.7–5.12	0.25	4.25	0.81	2.30–6.21	0.19
SOC (g/kg soil)	78.90	18.49	47.50–117.58	0.23	77.94	24.63	45.88–153.89	0.32
TN (g/kg soil)	6.03	1.38	3.65–9.26	0.23	5.17	1.28	3.27–8.82	0.25
C:N (g/g)	13.08	0.61	11.76–15.45	0.05	14.92	1.30	12.69–18.02	0.09
BD (g/cm^3^)	0.71	0.138	0.42–0.96	0.19	0.69	0.121	0.49–1.00	0.17
LAI (m^2^/m^2^)	3.50	0.60	2.60–4.90	0.17	2.96	0.30	2.41–3.68	0.10
Averaged SWC (cm^3^ cm^−3^)	0.31	0.0495	0.233–0.437	0.16	0.28	0.045	0.215–0.421	0.16
Seasonal CV of T_5_	0.27	0.02	0.22–0.30	0.08	0.32	0.02	0.28–0.38	0.07
Seasonal CV of SWC	0.21	0.04	0.14–0.30	0.20	0.17	0.05	0.07–0.30	0.29
LFOC (g/kg soil)	30.55	12.22	16.85–64.17	0.40	28.57	20.53	7.53–101.17	0.72
*L*_LFOC_ (g/g)	0.69	0.43	0.31–2.58	0.62	0.64	0.49	0.13–2.12	0.77
FR (g/m^2^)	223.40	76.80	31.04–330.94	0.34	164.45	61.07	69.45–298.32	0.37
NCP (m^3^/m^3^)	0.084	0.031	0.015–0.14	0.365	0.097	0.032	0.045–0.18	0.325

a S.D.: standard deviation; CV: coefficient of variance; R_S_: soil respiration; SWC: soil water content; TOC: total organic carbon; TN: total nitrogen; LFOC: light fraction organic carbon; FR: fine root biomass; BD: bulk density; LAI: leaf area index; NCP: non-capillary porosity. *n = *35. The soil respiration rates R_S_ and SWC in this table were averaged over the 12 (OF) or 13 (PP) measurement campaigns.

### Spatial Variation of Q_10_ Values

**Figure 3:**
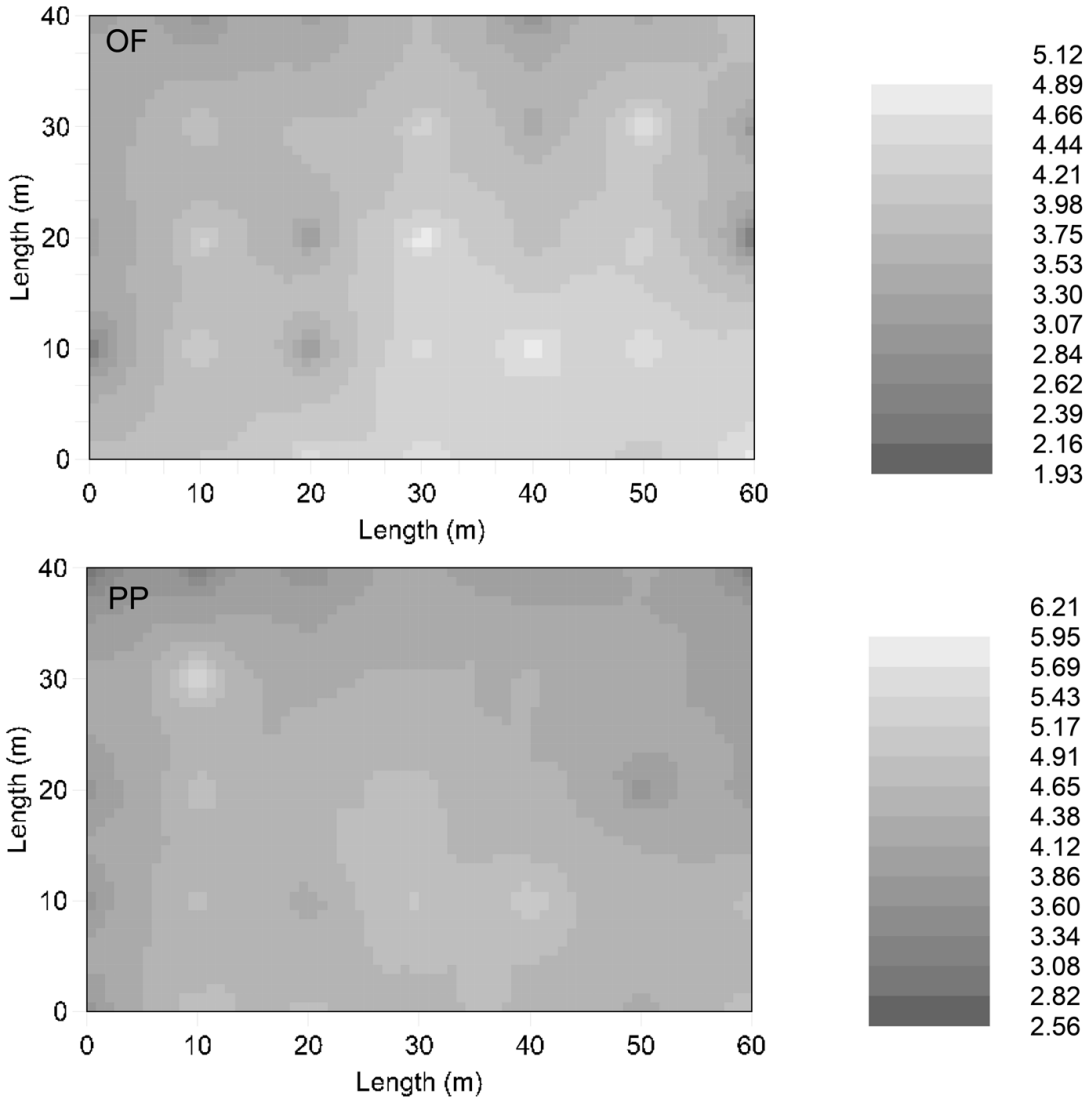
Isarithmic maps of the Q_10_ in the 10-m grids of OF and PP are shown in the top and bottom panels respectively, interpolations were done by the inverse distance weighting method. White areas indicate high values and dark areas indicate low values.

Exponential equation well described the relationship between R_S_ and T_5_ for each subplot, and all the correlations were significant at the P<0.05 (R^2^>0.34) level. The Q_10_ values varied considerably among subplots, ranging from 1.7 to 5.12 and 2.3 to 6.21 for the OF and the PP, respectively (Table 1). Among the Q_10_ values, 37.1% and 48.6% of them were between 4 and 5 for the OF and the PP, respectively. Spatial distribution of Q_10_ values for both forests are shown in Figure 3. According to our power calculation, the number of measurements required to estimate the Q_10_ of R_S_ per stand within 10% or 20% of its actual value at the 0.05 probability level are 26 and 6 for OF, respectively, and 15 and 4 for PP. Geostatistical analyses showed that Q_10_ values had no spatial autocorrelation (Fig. 1e, 2j). The absence of autocorrelations among Q_10_ values and soil parameters allowed us to treat our measurement locations as independent samples for inferential statistics.

### Controls on Q_10_ Variation

In PP, both FR and NCP were positively correlated with the Q_10_ values, while CV of T_5_ was negatively correlated with the Q_10_ values (Table 2). In OF, we found a significantly positive correlation between *L_LFOC_* and the Q_10_ values (*P* = 0.038; Table 2). Regardless of forest type, *L_LFOC_* and NCP were positively correlated, while SWC was negatively correlated with Q_10_ values (Table 2). No significant correlations between seasonal CV of SWC and Q_10_ were found for either forest or pooled data of two forests (Table 2). Significantly different Q_10_ values between forests was found (*F* = 4.517, *P* = 0.037; Table 3). However, significant difference in Q_10_ values between OF and PP disappeared when SWC or NCP was included as a co-variable in the GLM (Table 3).

**Table 2:** Pearson correlation coefficients between Q_10_ and variables in spatially.

Independent Variables	Pine plantation		Oak forest		Pooled data of two forests	
	R	Sig. (2-tailed)	R	Sig. (2-tailed)	R	Sig. (2-tailed)
LFOC	0.178	0.306	0.161	0.355	0.142	0.241
*L_LFOC_*	0.290	0.091	**0.351**	**0.038**	**0.293**	**0.014**
FR	**0.497**	**0.002**	0.240	0.165	0.207	0.086
SWC	−0.213	0.219	−0.246	0.155	−**0.290**	**0.015**
NCP	**0.369**	**0.029**	0.282	0.101	**0.355**	**0.003**
CV of T_5_	−**0.426**	**0.011**	−0.245	0.157	−0.010	0.932
CV of SWC	−0.053	0.762	−0.112	0.521	−0.169	0.161

Abbreviations see Table 1. n = 35 for each forest, n = 70 for pooled data of two forest types. The SWC in this table were averaged over the 12 (OF) or 13 (PP) measurement campaigns.

**Table 3:** General Linear Models for examine forest type effect on Q_10_ values, where F test was conducted. *L_LFOC_*, FR, SWC (averaged over 12 or 13 measurement campaigns), and NCP were taken as co-variables of the GLM respectively to examine which factor could exert influence on Q_10_ value difference between forest.

Variable type	Variables	*F* values	Sig.
Co variable	None	–	–
Fixed variable	Forest type	4.517	**0.037**
Co variable	*L_LFOC_*	7.539	**0.008**
Fixed variable	Forest type	5.689	**0.020**
Co variable	*FR*	8.965	**0.004**
Fixed variable	Forest type	10.548	**0.002**
Co variable	SWC	3.8	**0.055**
Fixed variable	Forest type	2.14	0.148
Co variable	NCP	7.7	**0.007**
Fixed variable	Forest type	2.62	0.11

Abbreviations see Table 1. None: No co-variable.

For all tests, df = 1 for fixed variable and co variables, and df = 67 for error.

## Discussion

### Spatial Variation of Q_10_ Values within Plots

Although the average Q_10_ values (3.80 and 4.25 for the OF and the PP) in this study was within the range of Q_10_ values reported in other temperate forests [42], [43], there was a large variation in Q_10_ values between subplots, such as 1.7–5.12 for the OF and 2.3–6.21 for the PP (see Table 1). Spatial variability in Q_10_ was also reported in a managed Ponderosa pine *(Pinus ponderosa)* forest (1.2–2.5; Xu and Qi [14]) and in a Japanese cedar *(Cryptomeria japonica)* plantation (1.3–3.2; Ohashi and Gyokusen [29]). This large variation of Q_10_ values among subplots suggests a potential risk of bias estimation of the soil respiration at a plot scale, which has not been adequately addressed. Similar estimates for soil respiration sampling have also been made in other studies. It was recommended to measure at least eight locations to stay within 20% of its actual value at the 95% confidence level in a mature beech forest [44]. Saiz et al. [45] also suggested that the sampling strategy of 30 sampling points per stand was adequate to obtain an average rate of soil respiration within 20% of its actual value at the 95% confidence level in four Sitka spruce stands.

### Controlling Factors on Q_10_ Variance

High spatial variance in soil moisture was found in both stands for most sampling dates (Figure 2), which could be attributed to the microtopography, the high spatial variability of soil organic matter content [34] and of root distribution (e.g. we found a significant negative correlation between SWC and fine root biomass *R^2^* = 0.16, *P* = 0.021, n = 35). Such a short scale soil moisture spatial variation have also been reported in other forests [29], [46], [47]. We even found a slight spatial autocorrelation for soil moisture (Fig. 1 d, i). It was reported that the high spatial variance of soil moisture exerted significant negative impact on soil respiration rate [34]. However, spatially, no significant impacts of soil moisture on Q_10_ values were found for PP and OF (Table 2).

In our study, all the subplots experienced similar seasonal fluctuations of soil temperature and moisture even though their magnitudes were different (Fig. 2). So we expect that there could be no obvious influence of different microclimate fluctuation on Q_10_ calculation at a given plot level in this study. However, the above mentioned influence was still found in PP where seasonal CV of T_5_ correlated significantly with Q_10_ values (Table 2). Nevertheless, microclimate fluctuation difference can not fully explain the spatial variability of Q_10_ values since no similar significant correlations were found in OF or when we pooled data together for all measurements regardless of forest types (Table 2). Therefore, we posit that the spatial variation of Q_10_ values among subplots should be associated with other inherent characteristics of each subplot, i.e, spatial differences in substrate availability as suggested by [15]. Gershenson et al. [16] also found a positive relationship between substrate availability and temperature sensitivity.

In our study, fine root biomass well explained the Q_10_ variance in PP, and was marginally significantly correlated with Q_10_ when we pooled data of all forest types (Table 2). Since fine roots are associated with the fast turnover carbon pool [48]–[50], the positive linear correlation between Q_10_ and FR implied the positive relationship between Q_10_ and lability of the substrate. It was also reported that Q_10_ values may be related to seasonal change in autotrophic respiration [17]. The correlations between fine root biomass and Q_10_ may also imply there exists a connection between Q_10_ and autotrophic respiration, i.e., the higher autotrophic respiration was coincided with the higher fine root biomass in the subplots. This inference was supported by our previous study as we found a similar positive correlation between FR and R_S_ [34].

Light fraction organic carbon (LFOC), which has been widely recognized as a labile carbon indicator [51], [52], is comprised largely of incompletely decomposed organic residues with turnover times of years to decades [53], thus the concentration of LFOC can indicate substrate supply quantity to some extent [34], [54], [55]. There was no correlation found between Q_10_ and labile organic carbon concentration (LFOC) as reflected by light fractionation (Table 2). Nevertheless, significant correlations between carbon pool lability (*L_LFOC_*) and Q_10_ were found in OF as well as when we pooled data together from all forest types (Table 2). This demonstrated that the carbon pool lability as reflected by light fractionation, which can partly stand for SOM quality [41], may exert more impact on Q_10_ values compared to the concentration of LFOC. This indicates a connection between the spatial distribution of SOM quality and the apparent Q_10_ as we speculated.

Multi-pool soil C models have been employed to simulate changes in soil C stocks as a single, homogeneous soil C pool [56]–[58], but the same Q_10_ value for different carbon fractions have still been applied. With increasing the understanding of temperature sensitivity of different soil organic carbon fractions [7]–[9]. Our findings on the connection between Q_10_ values and C availability among subplots suggest that different Q_10_ values corresponding to carbon fractions with different turn over times should be incorporated into soil carbon models.

### Q_10_ Values between Stands

In our study, Q_10_ values were significantly higher in the PP than that in the OF (Table 3), which is consistent with Wang et al.’s [59] findings in Korean pine plantation vs. *Mongolian* oak forest. Although we found significant correlations between *L_LFOC_* and FR with Q_10_ values, GLM showed that both *L_LFOC_* and FR can not explain why the higher Q_10_ occurred in the PP rather than in the OF (Table 3). No significant difference in Q_10_ values was found between PP and OF when averaged SWC was included as co-variables in GLM, but GLM showed a marginally significant correlation between averaged SWC and Q_10_. This implied that different soil moisture conditions accounted for different apparent Q_10_ values in the studied forests. Higher water content could impede O_2_ diffusion, thereby reducing decomposition rates and microbial production of CO_2_. In this case, the temperature response of CO_2_ efflux would be lower (i.e. a lower Q_10_ value) in wetter subplots than in dryer subplots, implying that the temperature response of CO_2_ efflux would be lesser in wet years than in dry years as Davidson et al [43] reported.

Furthermore, we speculate that effects of soil moisture conditions on Q_10_ may be partly attributed to different soil physical characteristics, such as the soil non-capillary porosity, which is an important factor in relation to soil gas diffusion. This was confirmed by our analysis, which showed that there was no significant difference in Q_10_ values between PP and OF when NCP was included as a co-variable, while there was a significant positive correlation between the spatial distribution of NCP and Q_10_ values (Table 3). This also indicated that the difference in NCP between two forests resulted in the difference in Q_10_ values. Similarly, a weak spatial correlation between hardness (related to soil porosity) of the A layer and Q_10_ variation was reported by Ohashi et al. [29]. Conant et al. [9] recently also suggested that the physico-chemical protection from decomposition of organic matter (OM) will affect temperature response of SOM. A negative correlation between averaged SWC and NCP (*R* = −0.306, *P* = 0.01) in this study regardless of forest type also suggested that there was an interaction between soil moisture and porosity. Soil porosity could exert intense impacts on temperature sensitivity of R_S_ in combination with soil moisture condition. Therefore, lower Q_10_ values in the OF compared to that in the PP may have been partly caused by the higher soil moisture or lower NCP.

In contrast, Xu and Qi [14] reported a positive correlation between Q_10_ values and soil moisture, with SWC values range from 10% to 24%. In our study, however, SWC values were 0.23–0.389 m/m^3^ for the PP and 0.241–0.451 m/m^3^ for the OF, respectively, which was higher than that reported by Xu and Qi [14]. This implies that there is a complex relationship between Q_10_ and soil moisture, which may result in contrasting effects. A marginal critical soil moisture condition may exist which determines a positive or negative relationship between Q_10_ and soil moisture.

### Conclusions

High spatial variances in apparent Q_10_ values were found for both forests. Parameters related to substrate availability and gas diffusion both exerted significant impact on the spatial variation of Q_10_ values within each stand. Higher Q_10_ values in the PP compared to the OF were also found, which could be attributed to the difference in soil moisture conditions or NCP, rather than substrate availability. Our results suggested that the R_S_ estimation at stand level could be improved through considering the spatial variation of Q_10_ values and its influencing factors.
